# Web Alert: Marine Microbial Natural Products: An annotated selection of World Wide Web sites relevant to the topics in microbial biotechnology

**DOI:** 10.1111/1751-7915.14363

**Published:** 2023-10-31

**Authors:** Lawrence P. Wackett

**Affiliations:** ^1^ McKnight Professor, Department of Biochemistry, Molecular Biology & Biophysics BioTechnology Institute, University of Minnesota St. Paul Minnesota USA

CMNPD Database

https://www.cmnpd.org

The Comprehensive Marine Natural Products Database (CMNPD) is an open‐access resource focussed on marine natural products research.

Screening using the CMNPD

https://www.mdpi.com/2075‐1729/13/6/1298

This paper describes *in silico* screening methods for identifying acetylcholinesterase inhibitors using the Comprehensive Marine Natural Products Database (CMNPD).

MarinLit

https://marinlit.rsc.org

MarinLit is a site dedicated to literature and compound information related to marine natural products research.

Marine natural product databases

https://onlinelibrary.wiley.com/doi/full/10.1002/biot.201800607

This review article highlights dedicated and tangentially related databases that could prove useful for those studying marine natural products. It focusses on freely available resources.

PeruNPDB

https://www.biorxiv.org/content/10.1101/2023.01.15.524152v1

This preprint describes an effort to focus on local marine natural products of Peru. Similar efforts are ongoing for Mexico, Brazil, India and China.

Marine natural products in clinical use

https://pubmed.ncbi.nlm.nih.gov/36005531/

Only a limited number of marine natural products reach the clinical stage of development, and this review focusses on those.

Antifouling marine natural products

https://www.frontiersin.org/articles/10.3389/fmars.2022.858757/full

Society invests considerable resources in preventing the build‐up of marine life on ships, harbour docks and offshore installations, often using toxic agents such as tributyltin. This article advocates for the use of biological controls to the problem.

Marine natural products at Scripps

https://scripps.ucsd.edu/research/topics/natural‐products‐chemistry

Studies on marine natural products are highlighted by this webpage offered by the Scripps Institution of Oceanography and the University of California at San Diego.

Marine natural products catalogue

https://www.medchemexpress.com/NaturalProducts/marine‐natural‐products.html

This commercial site offers a collection of marine natural products that may be purchased for screening purposes.

Marine natural products GRC

https://www.grc.org/marine‐natural‐products‐conference/2024/

Gordon Research Conferences discuss cutting edge research in different fields and this one to be held in 2024 focusses on marine natural products.

Marine natural product databases

https://d4‐pharma.com/marine‐natural‐products‐database‐for‐drug‐discovery/

This blog post describes the creation of a data compilation from more than 20,000 articles dealing with marine natural products.

Sustainable production of marine natural products

https://www.frontiersin.org/articles/10.3389/fmars.2023.1188090/full

This is an Editorial article highlighting different papers submitted for a *Frontiers* special issue on marine biotechnology and bioproducts.

Metagenomics for marine secondary products

https://journals.asm.org/doi/10.1128/spectrum.01501‐23

In this report, long‐read DNA sequencing was used to determine the biosynthetic capacity of prokaryotes in surface waters of the Yellow Sea.

Marine natural product tool

https://www.biorxiv.org/content/10.1101/2021.01.20.427441v1

This preprint details the development of a toolbox for identifying a broad range of biosynthetic gene cluster domains in metagenomic data.

Secondary products in the Arctic Ocean

https://www.microbiologyresearch.org/docserver/fulltext/mgen/7/12/mgen000731.pdf?expires=1697161491&id=id&accname=guest&checksum=C11D8730189C277E67AF0FD38348586D

Here is reported metagenomic sequencing of Arctic Ocean planktonic microbiomes and mining for novel biosynthetic gene clusters of natural products.

Biosynthetic potential of the global ocean

https://www.nature.com/articles/s41586‐022‐04862‐3

This study examined around 10,000 microbial genomes from marine sources that yielded approximately 40,000 biosynthetic gene cluster, most of which were characterized as being novel.

From ocean to medicine

https://www.ncbi.nlm.nih.gov/pmc/articles/PMC7460513/

This review article in the journal *Antibiotics* summarized reports appearing after 2014 and dealing with marine bacteria and their utility for the production of novel natural products.

